# A case report of a 5 mm port site hernia that occurred at the drain insertion site after laparoscopic right nephrectomy

**DOI:** 10.1093/jscr/rjae251

**Published:** 2024-04-24

**Authors:** Yoshiaki Kawamura, Takato Uchida, Tatsuya Umemoto, Nobuyuki Nakajima, Masahiro Nitta, Masanori Hasegawa, Sunao Shoji

**Affiliations:** Department of Urology, Tokai University School of Medicine, Isehara, Kanagawa 259-1193, Japan; Department of Urology, Tokai University School of Medicine, Isehara, Kanagawa 259-1193, Japan; Department of Urology, Tokai University School of Medicine, Isehara, Kanagawa 259-1193, Japan; Department of Urology, Tokai University School of Medicine, Isehara, Kanagawa 259-1193, Japan; Department of Urology, Tokai University School of Medicine, Isehara, Kanagawa 259-1193, Japan; Department of Urology, Tokai University School of Medicine, Isehara, Kanagawa 259-1193, Japan; Department of Urology, Tokai University School of Medicine, Isehara, Kanagawa 259-1193, Japan

**Keywords:** laparoscopic surgery, nephrectomy, port site hernia, steroids, aged, 80 and over, case report

## Abstract

A 5 mm port site hernia during laparoscopic surgery is rarer than a 12 mm port site hernia. Here, we report the case of a 5 mm port site hernia in an 85-year-old woman who underwent long-term steroid therapy and laparoscopic right nephrectomy. There was also a hernia at the port site where the drain was placed. Due to the 5 mm port at the drain removal site, fascial suturing was impossible after removal of the drain, and countermeasures were difficult. However, we believe that patients at a higher risk of port need suturing wound patients like this and should be carefully observed.

## Introduction

Laparoscopic surgery is now standard in urological surgery, and robot-assisted laparoscopic surgery is the mainstay. Port-site hernia is a complication of laparoscopic surgery, but its frequency is not high, with reports varying from 0.2% to 3.1% [1–4]. A 5 mm port site hernia is rare. In addition, hernias are more common in port holes of 5 mm or even rarer. Here, we report the case of a port site herniation in a 5 mm port hole where a drain was placed after laparoscopic right nephrectomy.

## Case report

**Figure 1:**
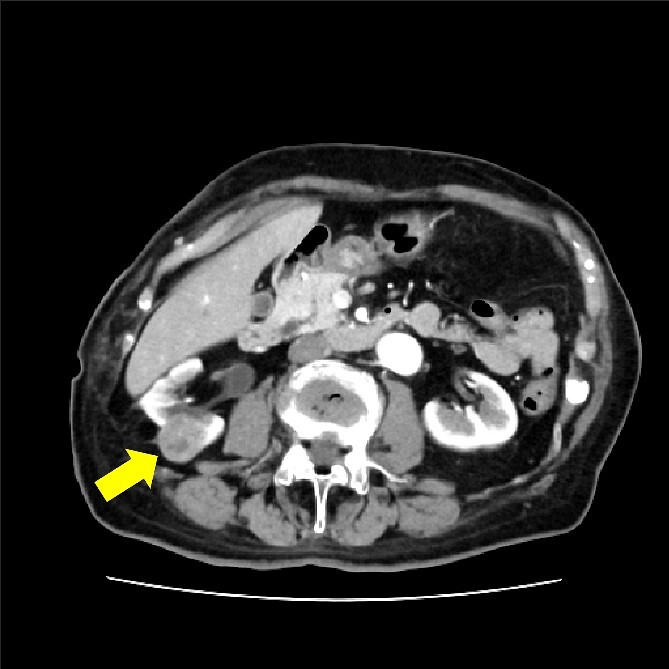
CT showing 30 mm right renal cell carcinoma. The arrow shows the tumor.

The patient was an 85-year-old woman with two pregnancies and two births. Her body mass index was 25 kg/m^2^. She had a history of hypertension, rheumatism, and open right pyeloplasty performed for stenosis of the right renal pelvic ureteric junction. A tumor in the right kidney was detected on an abdominal ultrasound examination and computed tomography (CT); the tumor size was about 30 mm (Fig. 1). A transperitoneal laparoscopic nephrectomy was performed. The operation time was 1 h and 52 min, and the blood loss was 10 ml. The surgery was completed without complications.

**Figure 2:**
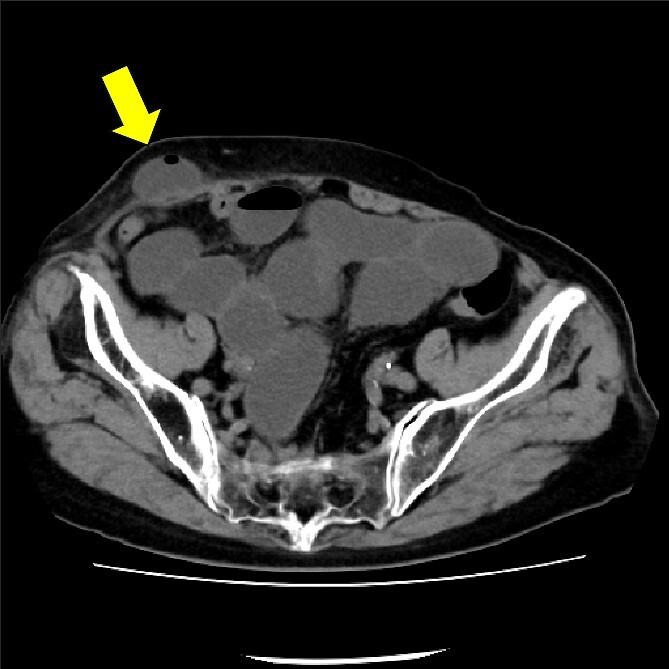
CT showing small intestine penetrating fascia of rectus abdominis. The arrow shows the incarcerated small intestine.

**Figure 3:**
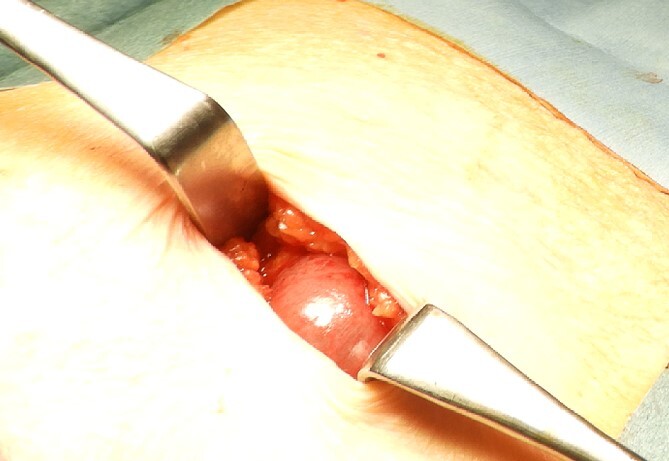
Small intestine incarcerated in the fascia of rectus abdominis.

**Figure 4:**
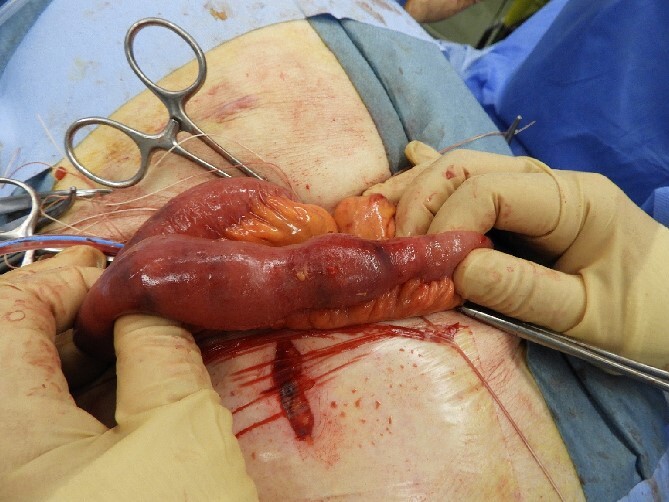
Slightly discolored small intestine.

The drain was removed on postoperative day 2. On the night of the third postoperative day, the patient vomited. On the morning of the fourth postoperative day, mild abdominal pain and a ping-pong ball-sized bulge and induration were found near the surgical scar of the 5 mm port where the drain had been placed. CT confirmed an incarcerated small intestine and ileus in the 5 mm port scar where the drain had been placed (Fig. 2). Ileus removal was performed on the same day. First, the dilated wound for nephrectomy was opened, and the inside of the abdominal cavity was observed. A hanging small intestine was observed just below the 5 mm port (Fig. 3). Since it could not be conquered manually, a 2 cm vertical incision was made on the 5 mm port wound. The small intestine penetrated the anterior layer of the rectus abdominis muscle, so the fascia was carefully incised to avoid damaging the small intestine. The small intestine was returned to the abdominal cavity. The small intestine was slightly discolored (Fig. 4), but after discussion with the gastrointestinal surgeon, it was determined that it was not necrotic. No small intestine resection was performed. The wound was carefully closed using fascial sutures to prevent recurrence. The fascia was weak, likely because this patient was taking steroids. The ileus has not recurred since then. The pathological results indicated a maximum diameter of 30 mm, consistent with papillary renal cell carcinoma, classified as pT1a G3 > G2, with negative margins.

## Discussion

A 5 mm port site hernia is rarer than a 10 mm or 12 mm port site hernia [5–8]. The largest number of 5 mm cases was published by Nezhat [3] in 1997 for 5 of 11 trocar-site hernias in a retrospective case review of ~5300 patients [8]. There are almost no reports of a 5 mm port site hernia in urological laparoscopic surgery, both domestically and internationally. Port site hernias are divided into the early type, in which the hernia contents penetrate the peritoneum, and the delayed type, in which the peritoneum becomes a hernial sac. The early type often develops within a few days to 2 weeks after surgery, and symptoms of intestinal obstruction occur rapidly.

Possible causes of port-site hernias include fascial damage due to the port being inserted at an acute angle rather than at a right angle, fascial damage due to multiple punctures, weakness of the abdominal wall including the fascia, peritoneal rupture due to sudden deflation, and intestinal collapse. Infection, fascial non-closure, wound infection, tissue at the time of drain removal, intestinal retraction, obese body shape, diabetes, postpartum, and older age are risk factors [4]. In this case, the port position was lateral to the rectus abdominis muscle and inserted perpendicular to the abdominal wall. Furthermore, steroid use has been cited as a predictive factor for the development of abdominal wall hernia [9]. Impaired wound healing may also be an adverse effect of long-term steroid administration. In this case, the port was inserted lateral to the rectus abdominis muscle and perpendicular to the abdominal wall. This case was thought to be caused by retraction of the intestine from the drain (15 Fr) removal site, advanced age, postpartum, and weakness of the abdominal wall tissue (due to long-term oral steroid use). Although fascial suturing of the entire wound is ideal for preventing a port site hernia, it is impossible at the site where the drain is removed. However, even if laparoscopic surgery involves a small wound and there is a high risk to the patient, we should close the fascia, and the patient should be closely observed for several days after the drain is removed.

In conclusion, we experienced a 5 mm port site hernia after laparoscopic urological surgery. Taking measures to prevent hernia development in the port hole of the drain-indwelling site is difficult. However, we believe that careful monitoring of cases with a high risk of hernia development is necessary.
